# Factors associated with intensive care admission in patients with lung cancer: a population-based observational study of 26, 731 patients

**DOI:** 10.1186/s12890-020-1071-8

**Published:** 2020-02-07

**Authors:** Kathryn Puxty, Christopher H. Grant, Philip McLoone, Billy Sloan, Tara Quasim, Kate Hulse, David S. Morrison

**Affiliations:** 1 Academic department of Anaesthesia, Critical Care and Pain, College of Medical, Veterinary and Life Sciences, University of Glasgow, Glasgow, UK; 2 Department of Public Health, College of Medical, Veterinary and Life Sciences, University of Glasgow, Glasgow, UK

## Abstract

**Background:**

Lung cancer is the most common cause of cancer related death worldwide and survival is poor. Patients with lung cancer may develop a critical illness, but it is unclear what features are associated with an Intensive Care Unit (ICU) admission.

**Methods:**

This retrospective, observational, population-based study of linked cancer registration, ICU, hospital discharge and mortality data described the factors associated with ICU admission in patients with lung cancer. The cohort comprised all incident cases of adult lung cancer diagnosed between 1st January 2000 and 31st December 2009 in the West of Scotland, UK, who were subsequently admitted to an ICU within 2 years of cancer diagnosis. Multiple logistic regression was used to determine factors associated with admission.

**Results:**

26,731 incident cases of lung cancer were diagnosed with 398 (1.5%) patients admitted to an ICU. Patients were most commonly admitted with respiratory conditions and there was a high rate of invasive mechanical ventilation. ICU, in-hospital and six-month survival were 58.5, 42.0 and 31.2%, respectively. Surgical treatment of lung cancer increased the odds of ICU admission (OR 7.23 (5.14–10.2)). Odds of admission to ICU were reduced with older age (75-80 years OR 0.69 (0.49–0.94), > 80 years OR 0.21 (0.12–0.37)), female gender (OR 0.73 (0.59–0.90)) and radiotherapy (OR 0.54 (0.39–0.73)) or chemotherapy treatment (OR 0.52 (0.38–0.70)).

**Conclusion:**

1.5% of patients diagnosed with lung cancer are admitted to an ICU but both short term and long term survival was poor. Factors associated with ICU admission included age < 75 years, male gender and surgical treatment of cancer

## Background

Lung cancer is the most common cancer and the leading cause of cancer related death worldwide [1]. Due to the nature of the disease and the aggressive treatments often employed, patients with lung cancer may develop a critical illness such that they require admission to ICU for invasive monitoring or treatment [2]. Of all the individual cancer types, lung cancer has one of the poorest survival after an ICU admission and has been demonstrated to have a high ICU and in-hospital mortality [3–5]. A systematic review of published outcomes for patients with solid tumours admitted to ICU described an average ICU mortality of 40.1% for patients with lung cancer, the highest of all the individual tumour types described [3]. However, lung cancer is one of the commonest tumour types admitted to ICU [2, 6, 7].

The proportion of cancer patients that are admitted to ICU is steadily increasing with one US hospital describing a two-fold increase in the number of lung cancer patients admitted to ICU over a ten-year period [8]. A wide variety of factors have been reported to be independently associated with short term mortality, the majority being related to the severity of the acute episode of illness requiring critical care and degree of organ dysfunction. However, no previously published studies have attempted to describe which factors are associated with ICU admission for these patients.

The aim of this study was to describe the rate of critical illness resulting in ICU admission among patients with lung cancer and the factors associated with this. We performed a population-based study using linked cancer registry, hospitalisation, ICU audit and death records to determine the risks after a cancer diagnosis.

## Methods

### Study design, Population & Setting

We conducted a retrospective, observational, multi-centre, population-based study through an analysis of secondary data comprising linked cancer registration, hospital discharge, intensive care and mortality records. Full details are described elsewhere [2]. Patients resident in the West of Scotland who had a diagnosis of lung cancer (ICD-10 codes; C33, C34.0, C34.1, C34.2, C34.3, C34.8 and C34.9) on the Scottish Cancer Registry between 1st January 2000 and 31st December 2009 were included in this study. Small Cell Lung Cancers (SCLC) were classified as ICD-O morphology M 8041/3 to M 8045/9; all others were classified Non-Small Cell Lung Cancers (NSCLC). We investigated whether they had been admitted to one of the 16 general ICUs located in the region within 2 years of the date of cancer incidence up to 31/12/2011. The first admission to an ICU within 2 years following a diagnosis of lung cancer was identified and linked to the appropriate individual episode of hospital care. We used death and hospital discharge records to identify whether patients died during their hospital stay.

### Data sources and variables

The study used four linked data sets: the Scottish Cancer Registry, Scottish Morbidity Record 01, national death records and the Scottish Intensive Society Audit Group WardWatcher ICU database. WardWatcher collects data on patient demongraphics, admitting specialty, admission diagnosis, the Acute Physiology and Chronic Health Evaluation (APACHE) II scoring system, and the type of organ support. Organ support was defined as receipt of invasive mechanical ventilation, vasoactive drugs to provide cardiovascular support, or renal replacement therapy.

Socioeconomic status was measured using the *Scottish Index for Multiple Deprivation* (SIMD 2009 V2 Scotland) quintile, an aggregate measure used to identify small area concentrations of deprivation.

The SMR01 hospital discharge records were reviewed for all primary and secondary diagnoses in the 5 years preceding the date of lung cancer incidence. These ICD-9 or ICD-10 coded diagnoses were computed to determine the presence or absence of co-morbid disease according to the Charleson Comorbidity Index (CCI) through previously published coding algorithms [9, 10].

This study was approved by the West of Scotland Research and Ethics Committee. Approvals to use the data were obtained from the West of Scotland Critical Care Research Network, SICSAG, and the West of Scotland Cancer Surveillance Unit.

### Statistical analyses

Patients who were admitted to an ICU were compared to the remainder of the incident lung cancer cohort according to demographic, clinical and cancer-related variables. Summary statistics including mean and standard deviation or median and inter-quartile range were determined for continuous exposure variables accordingly. Categorical variables were summarised according to frequencies and proportions. Non-ordered categorical variables were analysed by a chi-squared test of association whilst ordinal variables were compared between the two groups by use of a chi-squared test for trend.

Factors associated with admission to ICU were estimated using a multivariable logistic regression model. The odds of survival were computed for each exposure variable using univariable analyses with 95% confidence intervals and *P* values. Variables found to have a potentially statistically significant alteration in the odds of survival (*P* < 0.3) in univariable analyses were included in an adjusted multivariable model. The cancer treatment modalities (surgery, radiotherapy, chemotherapy) were not included in any multivariable model due to potential collinearity with treatment intent. A *P* value of < 0.05 was used to identify statistically significant associations in multivariable modelling for ICU admission.

Statistical analyses were performed using *StataCorp 2011* (Stata Statistical Software: Release 12).

### Availability of data and materials

The authors do not have permission to share the data used for this article, however, the data can be accessed via application to the Information Services Division of NHS Scotland.

## Results

**Fig. 1:**
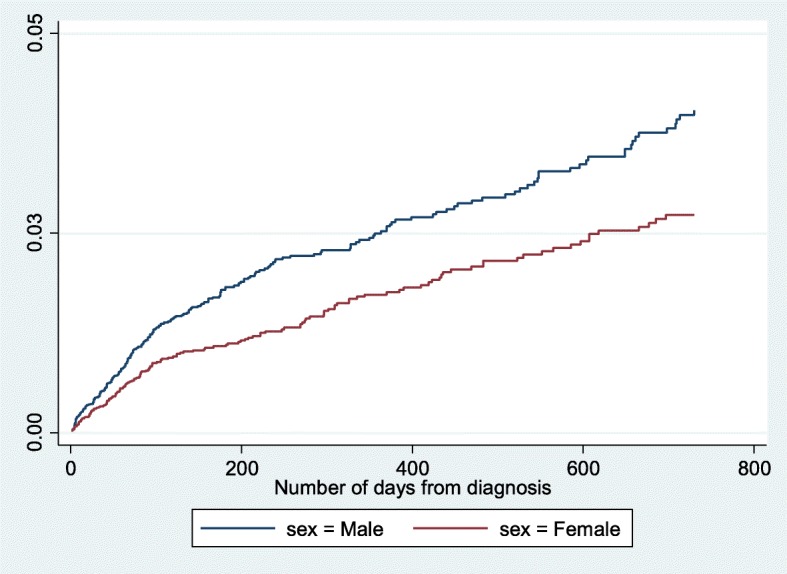
Cumulative hazard of admission to Intensive Care by time since cancer incidence date according to sex (log-rank test for equality of survivor functions Chi^2^ 15.22, *P* < 0.001)

There were 26,731 incident cases of primary lung cancer diagnosed during the study period of whom 398 patients (1.5%) were admitted to an ICU within 2 years of diagnosis. Figure 1 shows the cumulative hazard curve for time to admission to ICU in days according to sex. There appears to be a sharp rise in the hazard of ICU admission within the first 100 days following a diagnosis of lung cancer for both sexes. The median number of days from lung cancer diagnosis to admission was 52 (IQR 0–106). There were fewer ICU admissions amongst women observed than were expected.

Features pertaining to the ICU admission are detailed in Table 1. The majority of lung cancer patients admitted to ICU were following surgical hospitalisations. Common causes for admission to ICU included lower respiratory tract infection (27.4%), conditions directly related to lung cancer such as bronchial obstruction or bronchial haemorrhage (20.3%), and following lung resection surgery (15.3%). Organ support was frequently utilised with 40.1% of patients requiring multi-organ support. ICU and hospital median length of stay were 3 (IQR 1–6) and 10 days (IQR 1–19). The proportions of patients who survived ICU, hospital and six-months after hospital discharge were 58.5, 42.0 and 31.2%, respectively.

**Table 1:** Clinical features of Lung cancer patients admitted to ICU

Categories	*N* = 368 N (%)
Admission Diagnosis	
Related to lung cancer	81 (20.3)
Acute cardiac decompensation	43 (10.8)
Chest infection	109 (27.4)
Related to surgical treatment	61 (15.3)
Unrelated to lung cancer	104 (26.1)
Cardiac Arrest Precipitating Admission	
Prior CPR	29 (7.3)
No prior CPR	369 (92.7)
APACHE II Score	
8–12	20 (5.0)
13–17	67 (16.8)
18–22	77 (19.4)
23–27	74 (18.6)
≥ 28	55 (13.8)
Missing	105 (26.4)
Admission Specialty	
Medical	134 (33.7)
Surgical	264 (66.3)
Number of Organs Supported	
None	79 (19.9)
1	94 (23.6)
2	138 (34.7)
3	25 (6.3)
Not known	62 (15.6)
Receipt of Mechanical Ventilation	
No	99 (24.9)
Yes	282 (70.9)
Not known	17 (4.3)
Receipt of Renal Replacement Therapy	
No	322 (80.9)
Yes	27 (6.8)
Not known	49 (12.3)
Receipt of Vasoactive Agents	
No	183 (46.0)
Yes	182 (45.7)
Not known	33 (8.3)
Charlson Co-Morbidity Index	
0	239 (60.1)
≥ 1	159 (39.9)
Length of stay	
ICU	3 days (IQR 1–6)
Hospital	10 days (IQR 1–19)
Mortality	
ICU	165 (41.5)
Hospital	231 (58.0)
Six-month	274 (68.8)

Table 2 compares the 398 patients admitted to ICU within 2 years of diagnosis to the remaining 26,333 incident primary lung cancer cases. Patients admitted to ICU had a younger age of diagnosis than the non-ICU cohort. There was a larger proportion of males admitted to ICU (61.8%) than the remainder of the cohort (53.5%). The largest SIMD quintile represented in each group was the most deprived but deprivation was not associated with admission to ICU. Non-Small Cell Lung cancer was the commonest tumour type in both the ICU and non-ICU groups with a higher proportion in the ICU group (92% versus 84%). Comorbidity was common in both groups (39.9% ICU admissions vs. 35.8% of non-ICU admissions, p 0.09). Those admitted to an ICU were more likely to have undergone a surgical treatment for lung cancer with a curative treatment plan. Treatment with radiotherapy or chemotherapy occurred in a higher proportion of the non-ICU population.

**Table 2:** Features of lung cancer patients that are and are not admitted to Intensive Care

	ICU Admissions *N* = 398 n (%)	Non-admissions *N* = 26,333 n (%)	*P* value
Age at Incidence			
Mean (SD)	67 (SD 8.8)	71 (SD 10.3)	**< 0.001**
Sex			
Male	246 (61.8)	14,085 (53.5)	**0.001**
Female	152 (38.2)	12,248 (46.5)	
SIMD Quintile			
1 (Most deprived)	146 (36.7)	10,691 (40.6)	0.350^a^
2	106 (26.6)	6692 (25.4)	
3	73 (18.3)	4108 (15.6)	
4	43 (10.8)	2739 (10.4)	
5 (Most affluent)	30 (7.5)	2103 (8.0)	
Histology			
SCLC	32 (8.0)	4222 (16.0)	**< 0.001**
NSCLC	366 (92.0)	22,111 (84.0)	
Charlson Co-Morbidity Index			
0	239 (60.1)	16,894 (64.2)	0.090
≥ 1	159 (39.9)	9439 (35.8)	
Year of Cancer Incidence			
2000	43 (10.8)	2562 (9.7)	**0.004**^**a**^
2001	39 (9.8)	2389 (9.1)	
2002	42 (10.6)	2624 (10.0)	
2003	57 (14.3)	2600 (9.9)	
2004	35 (8.8)	2607 (9.9)	
2005	42 (10.6)	2609 (9.9)	
2006	38 (9.5)	2738 (10.4)	
2007	46 (11.6)	2716 (10.3)	
2008	35 (8.8)	2700 (10.3)	
2009	21 (5.3)	2788 (10.6)	
Treated with Surgery			
No	168 (42.2)	21,540 (81.8)	**< 0.001**
Yes	210 (52.8)	2505 (9.5)	
Not known	20 (5.0)	2288 (8.7)	
Treated with Radiotherapy			
No	280 (70.4)	15,510 (58.9)	**< 0.001**
Yes	53 (13.3)	7876 (29.9)	
Not known	65 (16.3)	2947 (11.2)	
Treated with Chemotherapy			
No	283 (71.1)	17,342 (65.9)	**< 0.001**
Yes	56 (14.1)	6468 (24.6)	
Not known	59 (14.8)	2523 (9.6)	
Therapy objectives			
Curative	140 (35.2)	2481 (9.4)	**< 0.001**
Palliative	184 (46.2)	20,657 (78.5)	
Not known	74 (18.6)	3195 (12.1)	

*SCLC* Small cell lung cancer, *NSCLC* Non-small cell lung cancer

Table 3 describes factors associated with ICU admission on univariable and multivariable logistic regression analysis. The largest effect was seen for patients receiving surgical treatment for cancer, with OR 7.23 (95% CI 5.14–10.2) for ICU admission, when compared to patients that did not receive surgical intervention. Unknown treatment intent was associated with ICU admission to a lesser extent (OR 1.54 (95% CI 1.08–2.20)). Increasing age, female gender and radiotherapy or chemotherapy treatment were all associated with a reduced odds of ICU admission in the multivariable model.

**Table 3:** Factors associated with ICU admission on univariable and multivariable logistic regression

	Univariable Odds Ratio (95% CI)	*P* value	Multivariable Odds Ratio (95% CI)	*P* value
Age at Incidence Quintile*				
15–62	1		1	
63–69	1.21 (0.93–1.57)	0.153	1.15 (0.88–1.51)	0.294
70–74	0.90 (0.67–1.20)	0.463	0.87 (0.64–1.17)	0.360
75–80	0.62 (0.46–0.85)	**0.003**	**0.69 (0.49–0.94)**	**0.019**
> 80	0.16 (0.09–0.28)	**< 0.001**	**0.21 (0.12–0.37)**	**< 0.001**
Sex				
Male	1		1	
Female	0.71 (0.58–0.87)	**0.001**	**0.73 (0.59–0.90)**	**0.003**
SIMD Quintile				
1 (Most deprived)	1		1	
2	1.16 (0.90–1.49)	0.249	1.16 (0.89–1.50)	0.269
3	1.30 (0.98–1.73)	0.068	**1.37 (1.03–1.83)**	**0.033**
4	1.15 (0.82–1.62)	0.425	1.31 (0.92–1.86)	0.132
5 (Most affluent)	1.04 (0.70–1.55)	0.829	1.08 (0.69–1.55)	0.696
Therapy Objectives				
Curative	1		1	
Palliative	0.16 (0.13–0.20)	**< 0.001**	1.08 (0.75–1.54)	0.682
Not known	0.41 (0.31–0.55)	**< 0.001**	**1.54 (1.08–2.20)**	**0.017**
Treated with Surgery				
No	1		1	
Yes	10.75 (8.7–13.2)	< 0.001	**7.23 (5.14–10.2)**	**< 0.001**
Not known	1.12 (0.70 - 1.79)	0.631	**0.50 (0.27–0.91)**	**0.025**
Treated with Radiotherapy				
No	1		1	
Yes	0.37 (0.28–0.50)	< 0.001	**0.54 (0.39–0.73)**	**< 0.001**
Not known	1.22 (0.93–1.60)	0.150	1.32 (0.78–2.24)	0.298
Treated with Chemotherapy				
No	1		1	
Yes	0.53 (0.40–0.71)	< 0.001	**0.52 (0.38–0.70)**	**< 0.001**
Not known	1.43 (1.08–1.90)	0.013	1.07 (0.62–1.84)	0.814
Charlson Co-morbidity Index				
0	1		1	
≥ 1	1.19 (0.97–1.46)	0.091	1.22 (0.99–1.51)	0.059

## Discussion

In a large inclusive cohort of lung cancer patients diagnosed over a ten-year period, we found that 1.5% were admitted to a general ICU within 2 years of diagnosis. ICU admission was greatest in the period shortly after cancer diagnosis and this is likely to reflect patients developing critical illness as a consequence of active cancer or its treatment.

### Clinical features in patients with lung cancer admitted to ICU

Reasons for ICU admission were predominantly related to respiratory conditions with lower respiratory tract infections and complications from the malignancy accounting for 27.4 and 20.3% of all admissions respectively. However, the ICU admission was entirely unrelated to the lung cancer diagnosis/ treatment in one in four ICU lung cancer patients. The majority of ICU patients with lung cancer required organ support, the most common modality being invasive ventilation (70.9%) reflecting the high incidence of respiratory disorders.

For patients admitted to ICU, mortality in ICU, hospital and six-months post admission were 41.5, 58.0 and 68.8%, respectively. Mortality in our study was relatively high when compared with previous multi-centre studies [11–13]. The largest study of this nature was performed on the American surveillance, epidemiology and end results- medicare registry (SEER), including nearly 50,000 patients. Hospital mortality was reported as 24%, significantly lower than that seen in this study. However, mortality at six-months was similar at 65% [13]. This registry-based study did not detail any of the features of the critical illness which has been demonstrated to be the largest determinant of short-term mortality [3]. Soares et al conducted the only prospective, multi-centre cohort study and reported ICU, in-hospital and six-month mortality of 28, 39 and 55% respectively. However, the proportion of subjects requiring mechanical ventilation was only 53% compared with the 79% observed in our study, suggesting that the burden of critical illness was less.(11).

### Differences between ICU and non-ICU groups

While the majority of patients with lung cancer were treated with palliative intent, a disproportionate number of patients in the ICU group had received curative treatment with the majority of patients receiving surgical intervention. This observation likely reflects clinician behaviour whereby patients with curative treatment intent are preferentially selected for ICU during an acute critical illness. However, this could also be partially attributable to post-operative complications in patients undergoing curative surgical procedures as the majority of ICU admissions were surgical in nature (66%) and ICU admission was directly related to cancer surgery in 15%.

### Factors associated with ICU admission in patients with lung cancer

Surgical treatment of cancer was found to be the strongest predictor of ICU admission with a seven-fold increase in odds of ICU admission compared with those not treated with surgery. This may be due to critical illness occurring at the time of surgery with one in eight ICU admissions were directly attributable to the surgical treatment. While it is possible that patients having received curative surgery were being considered better candidates for ICU, treatment intent was not associated with ICU admission on the multivariable model. Other treatment interventions influenced ICU admission by reducing the odds by approximately half in those who were treated with chemotherapy or radiotherapy compared with those who were not. As these patients are being admitted early after diagnosis they may not have had the opportunity to receive these treatment interventions prior to ICU admission.

Age of incidence was found to be a strong negative predictor of ICU admission with a reduction in the odds of admission with increasing age above 75 years that reduces further for those aged over 80 years. Previous work by Azoulay et al. demonstrated that the largest influence on a refusal for ICU admission in critically ill patients with cancer was an age of over 65 years [14]. Within the general ICU population, increasing age has been associated with poorer survival both in the short and longer term, and that this was particularly pronounced for patients aged over 75 years [15]. It seems unlikely that critical illness is less common with increasing age, particularly given the increased incidence of comorbidities encountered in an elderly population, rather that concerns about poor outcomes in the elderly patients swings the balance of benefit versus harm away from ICU admission.

Comorbidity was not associated with an increase in odds of ICU admission in the multivariable model. The study by Slatore et al. [16] demonstrated that comorbidity has been associated with poorer outcome after ICU admission in patients with lung cancer, however, our study suggests that this does not necessarily influence admission patterns.

Females had a statistically significant reduction in the odds of ICU admission compared to males (OR 0.75, 95% CI 0.61–0.92, *P* = 0.006). An increased incidence of critical illness in men has been described for patients with severe sepsis and trauma [17, 18]. The cause of this is not clear but may reflect differences in lifestyle choices, health behaviours or the impact of hormones on stress responses. Furthermore, it is possible that the gender difference may not reflect difference in rates of critical illness but a difference in preferences for end of life care. It is known from other studies that survival from lung cancer is poorer in men than women overall. Among those with a critical illness that we report, survival was also poorer in men than women. Given that a minority of lung cancer patients is admitted to an ICU (1.5%), we would conclude that both non-critically ill as well as critically ill female lung cancer patients have better survival than men.

### Strengths and weaknesses

Our study has a number of strengths. It is the first study to compare lung cancer patients admitted to an ICU with those who were not over a ten-year period. It utilised a large sample size and was conducted across multiple general rather than oncological ICUs, which may improve its generalisability. We used datasets with high levels of case ascertainment for cancer incidence, hospitalisation and deaths. Measurement bias was reduced through the use of objective scoring systems to capture acute illness and past medical history. The use of the SIMD provided a robust, multi-dimensional measure of socioeconomic status rather than relying on a single domain as a proxy for affluence as in previous studies [12, 13, 19]. Exposure variables from the ICU admission were collected and determined prospectively thereby reducing the likelihood of misclassification.

This study has several limitations. Large proportions of missing data precluded the use of information on ethnicity, cancer stage and pathological sub-division which could confound the observed associations. Furthermore, while the date of cancer diagnosis in all cases was prior to ICU admission, we could not determine the certainty of diagnosis at the point of entry to critical care which may affect decisions about whether to admit a patient. We accept that approaches to managing critical illness are likely to differ in patients receiving palliative care and while we adjusted for treatment objective (curative, palliative or unknown), we did not have information on palliative care involvement within the data sources utilised in this study.

## Conclusion

Lung cancer is the most common cause of cancer related death worldwide [1]. Our results highlight that only a small proportion of patients with lung cancer were subsequently admitted to an ICU within 2 years of diagnosis (1.5%). Over half of those who were admitted survived to ICU discharge with nearly a third surviving to six-months from admission. It is unclear why some factors that are usually associated with a better prognosis, such as younger age and surgical treatment, are associated with greater risks of ICU admission. This may represent physician decision making and further prospective research is needed to explore the clinical pathways prior to ICU admission.

## Data Availability

The data is available through application to the NHS Scotland Information Services Division.

## Abbreviations

APACHE: Acute Physiology and Chronic Health Evaluation
CCI: Charlson Co-morbidity Index
CI: Confidence Interval
HDU: High Dependency Unit
ICU: Intensive Care Unit
ICD: International Statistical Classification of Diseases and Related Health Problems
NSCLC: Non Small Cell Lung Cancer
OR: Odds Ratio
SCLC: Small Cell Lung Cancer
SICSAG: The Scottish Intensive Care Society Audit Group
SIMD: The Scottish Index for Multiple Deprivation
SMR 01: The Scottish Morbidity Record 01
